# Tailorable Burning Behavior of Ti14 Alloy by Controlling Semi-Solid Forging Temperature

**DOI:** 10.3390/ma9080697

**Published:** 2016-08-16

**Authors:** Yongnan Chen, Wenqing Yang, Haifei Zhan, Fengying Zhang, Yazhou Huo, Yongqing Zhao, Xuding Song, Yuantong Gu

**Affiliations:** 1 School of Materials Science and Engineering, Chang’an University, Xi’an 710064, China; winky_ywq@163.com (W.Y.); zhangfengying@chd.edu.cn (F.Z.); yazhouhuo@chd.edu.cn (Y.H.); 2 School of Chemistry, Physics and Mechanical Engineering, Queensland University of Technology (QUT), Brisbane, QLD 4001, Australia; yuantong.gu@qut.edu.au; 3 Northwest Institute for Non-Ferrous Metal Research, Xi’an 710016, China; trc@c-nin.com; 4 School of Construction Machinery, Chang’an University, Xi’an 710064, China; Songxd@chd.edu.cn; 5 School of Mechanical Manufacture and Automation, North University of China, Taiyuan 030051, China

**Keywords:** Titanium, semi-solid forging, temperature, microstructure, burning behavior

## Abstract

Semi-solid processing (SSP) is a popular near-net-shape forming technology for metals, while its application is still limited in titanium alloy mainly due to its low formability. Recent works showed that SSP could effectively enhance the formability and mechanical properties of titanium alloys. The processing parameters such as temperature and forging rate/ratio, are directly correlated with the microstructure, which endow the alloy with different chemical and physical properties. Specifically, as a key structural material for the advanced aero-engine, the burn resistant performance is a crucial requirement for the burn resistant titanium alloy. Thus, this work aims to assess the burning behavior of Ti14, a kind of burn resistant alloy, as forged at different semi-solid forging temperatures. The burning characteristics of the alloy are analyzed by a series of burning tests with different burning durations, velocities, and microstructures of burned sample. The results showed that the burning process is highly dependent on the forging temperature, due to the fact that higher temperatures would result in more Ti_2_Cu precipitate within grain and along grain boundaries. Such a microstructure hinders the transport of oxygen in the stable burning stage through the formation of a kind of oxygen isolation Cu-enriched layer under the burn product zone. This work suggests that the burning resistance of the alloy can be effectively tuned by controlling the temperature during the semi-solid forging process.

## 1. Introduction

Owing to the high strength and excellent corrosion resistance, titanium and titanium alloys have shown potential engineering applications, such as biomedical engineering, the chemistry industry, and aerospace [1]. However, the extensive applications of titanium and titanium alloys have been greatly limited for their poor formability (such as high deformation load and low thermal conductivity) and high processing cost [2]. In this regard, plenty of research efforts, such as advanced forging/rolling technologies [3,4], alloying with low-cost metal element [5], and heat treatment [6], have been devoted to improve the formability of titanium alloys. Semi-solid processing (SSP) is one of the representative techniques that are proposed to improve the formability of metal alloys, such as titanium alloys [7,8,9,10], aluminum alloys [11,12,13,14,15], magnesium alloys [16,17,18], and steel products [19,20,21]. Fundamentally, SSP is a processing of mushy or semi-solid metals or alloys, where the alloy consists of solid and liquid phases [22]. In such a state, the liquid component encloses the solid crystals, which allows the slips and the rotations of crystals, enhances the formability of metals, and reduces the processing load in the forming of complicated products [23].

To successfully apply the SSP technique, the alloy should have a definite solid-liquid region with a wide freezing range before processing. One crucial parameter is the solid-liquid fraction in the alloy, which largely determines the SSP temperature. Higher amounts of the solid fraction are usually preferred as they reduce the chance of volumetric defects, increase the stability of the material under its own weight, promote the smooth laminar flow of liquid, and also improve the surface quality and the internal structure of the formed components [24]. Previous works on the deformation behavior of the metals [7,25] have shown that a solid fraction between 75% and 95% is commonly suitable for SSP, such as semi-solid forging [26], rolling [17] and extrusion [16]. In addition to formability, the SSP temperature plays a critical role in the final microstructure of the alloys (which determines their chemical and physical properties), including the distribution and morphology of grain and precipitation during solidification. The study on the semi-solid deformation and forging behavior of aluminum alloys, such as Al-Si [27], and Al-Cu alloy [28], showed a high-temperature dependence on microstructure, especially on precipitation. Similar results were also reported by semi-solid extruding of magnum alloy [29] and steel [30] where, by controlling the temperature and composition, a high potential of semi-solid forging in improving the formability and mechanical properties of metal product was suggested.

For the SSP of titanium alloy, a burn-resistant Ti-Cu alloy (Ti14) with α+Ti_2_Cu phase structure designed for potential application in advanced engines, is considered to be suitable for semi-solid forging due to the low melting Ti_2_Cu phase (990 °C) and wide freezing [8,31,32]. The previous works on deformation and forging behavior of Ti14 alloy have demonstrated that the mechanical property was directly related to their microstructural characteristics, especially on morphology and the nature of Ti_2_Cu precipitates that depend on SSP temperature [33,34]. However, as a key structural material for advanced aero-engines, the influence of the forging temperature on the burn characteristic, which is a crucial requirement for burn resistant alloys, is still unknown. In the literature, plenty of works have assessed the factors that affect the burn characteristics of titanium alloys, such as the composition [35,36,37] and critical ignition boundary (such as oxygen partial pressure and surface pressure) [38,39,40]. However, none of the studied samples are forged at the semi-solid state. Therefore, this work is intended to acquire the burning behavior of the burn-resistant Ti14 alloy forged in the semi-solid state under different temperatures. For such a purpose, a modified direct current simulation burning (DCSB) method [40] was employed. Through the comprehensive analysis of the burning characteristics, e.g., flame height, duration, velocity, and burned sample structure, it is found that the burning behavior and burn resistant mechanism of Ti14 alloy has high dependence on the forged microstructure, which may consummate the basic research for semi-solid processing of burn-resistant titanium alloys.

## 2. Experimental Sections

### 2.1. Sample Preparation

The burn-resistant Ti14 alloy with α+Ti_2_Cu phase was selected for semi-solid forging. According to atomic emission spectroscopy, the exact composition of the extruded bar alloy was Ti-85%Cu-14%Al-0.3%Si-0.7% alloy. Since the melting point of Ti_2_Cu is 990 °C [7], which means that the Ti_2_Cu will change to liquid and the alloy will change to a semi-solid state when the deformation or testing temperature is over 990 °C. Three temperatures (1000 °C, 1050 °C, and 1100 °C) were selected in the solid + liquid (S + L) region to carry out the semi-solid forging. These temperatures were chosen to ensure smooth laminar flow of liquid during the deformation [7,33], as liquid segregation and leakage will occur for higher temperature [34] (due to the low thermal conductivity of titanium). The isothermal holding time for the alloy in the semi-solid state before forging was 10 min. The forging die was coated with the graphite lubricant before the forging, and heated to 1000 °C to avoid thermal losses during the forging. A radiation thermometer was used to measure the surface temperature of the specimen prior to the test, and the forging operations were conducted in a hydraulic press at a die speed of 500 mm/s [33]. Samples were subjected to 60% reduction at each forging temperature. After the forging, the alloy was immediately quenched in hot water to avoid microstructure coarsening due to the longer solidification time.

**Figure 1:**
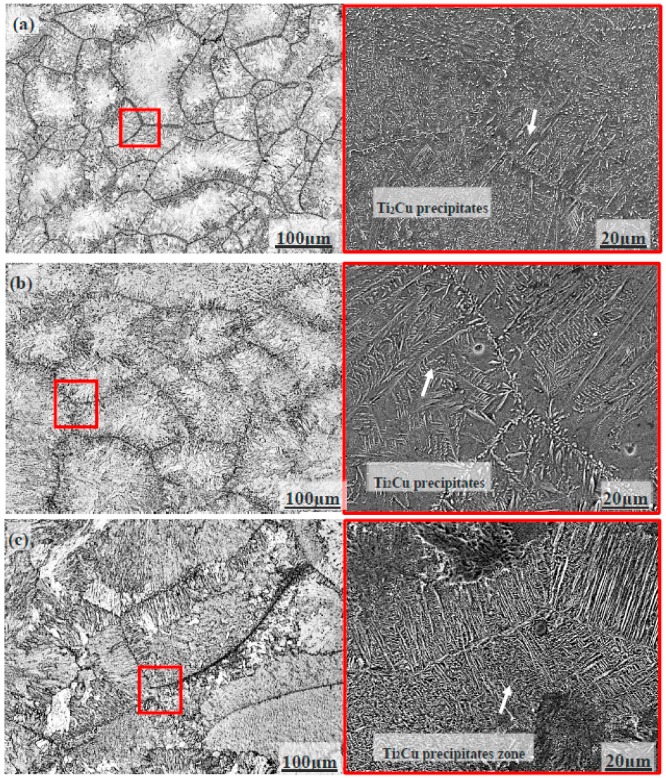
Microstructures of Ti14 alloy at the forging temperature of: (**a**) 1000 °C; (**b**) 1050 °C; and (**c**) 1100 °C. Right figures show the enlarged views of the grain boundaries as obtained from scanning electron microscope (SEM, JSM-6700, JEOL Company, Osaka, Japan) [33].

Figure 1 shows the microstructures of the alloy at different forging temperatures. As reported in our previous work [33], the forged alloy mainly consisted of α-Ti matrix and Ti_2_Cu precipitates. By estimating the accumulated Ti_2_Cu fraction around the grain boundary, the semi-solid forged Ti14 alloy possesses much higher percentage of Ti_2_Cu precipitates comparing with that of the solid forged sample. Increasing the semi-solid forging temperature, a higher percentage of Ti_2_Cu precipitates on grain boundary is observed, which is accompanied by a decreased percentage within grain. This fact leads to a slightly reduced mechanical property of the Ti14 alloy (tensile strength, yield strength, elongation, and ductility) at higher semi-solid forging temperature. However, comparing with the solid forged sample, the semi-solid forged sample has superior tensile strength and yield strength, as shown in Table 1.

### 2.2. Burning Test

**Figure 2:**
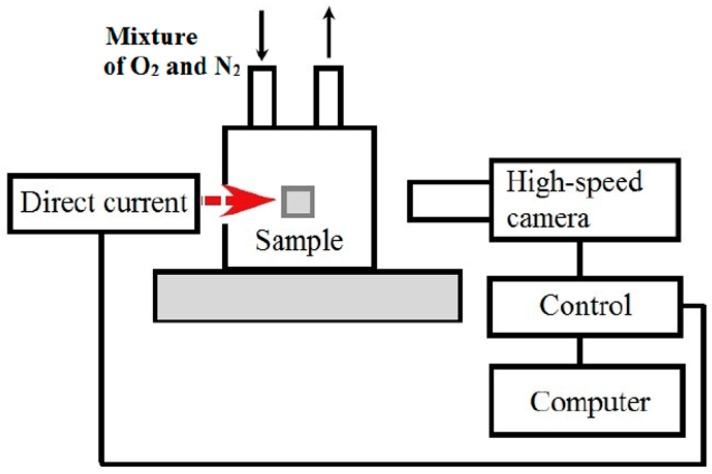
Schematic view of the burning experimental setup.

A modified direct current simulation burning (DCSB) method was employed to acquire the burning behavior of the Ti14 alloy [36,37], and the examined sample has a similar cuboid-shape (with a size of 5 × 10 × 20 mm^3^) for comparison purposes. As schematically shown in Figure 2, a direct current of 5A in a pre-mixed gas was used to ignite the sample [35], and the direct current was turned off immediately after successful ignition. In order to investigate the influence of gas condition on the burning characteristics, the pre-mixed gas (O_2_/N_2_) with the oxygen partial pressure (*C*_o_) from 20% (air condition) to 100% (oxygen condition) and flow velocity 15 m/s was controlled by the gas supply system (Paker F65). Among that, the burning of the titanium alloy mainly occurs in the air condition (*C*_o_ = 20%) [35,36,40], which is the major focus in this study. A digital high-speed video camera (Pco 1200 hs, PCO Company, Berlin, Germany) was used to record the burning behaviors of the alloy, including flame height, burning duration, and velocity. The images were taken at a frame interval of 500 μs and an exposure time of 10 μs. To avoid exceeding the light saturation level of the camera, neutral density filters were placed between the burning sample and the microscope. The temperatures were measured using both R thermocouples when ignition occurred. The thermocouple was placed in the center of samples. X-ray diffraction (XRD, XRD-1700, Bluker Company, Berlin, Germany) and energy dispersive X-ray spectroscopy (EDS, JSM-6700, JEOL Company, Osaka, Japan) microanalysis were used to investigate the burned products and chemical composition of selected regions of burned structure.

## 3. Results and Discussion

### 3.1. The General Burning Behavior

**Figure 3:**
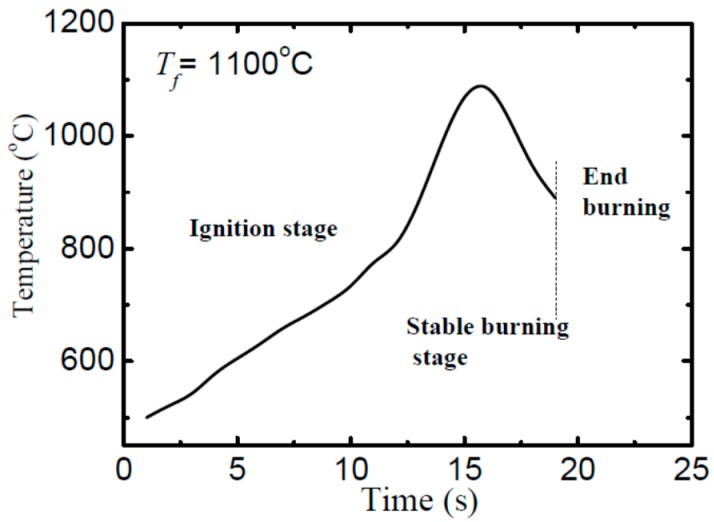
A representative temperature profile measured at the center of the sample (obtained from forging temperature of 1100 °C). A preheating process at 500 °C was carried out before burning test to accelerate the ignition.

The burning behavior of titanium alloy usually occurs as a two-stage process, ignition and stable burning stages [35]. The two stages are usually distinguished by the gradient change of the heat/temperature caused by different chemical reactions [40]. Such burning characteristics are commonly obtained from metals, such as lithium alloys [41], magnesium alloys [42], and particles of zinc [43] and titanium [44]. Figure 3 shows a representative temperature profile measured at the center of the sample during burning (from the alloy forged at a temperature of 1100 °C). To note that since the thermocouple was placed at the middle of the sample, the recorded temperature does not reflect the actual temperature at the burning site (due to the low conductivity of the alloy, ~21.9 W/mK at 300 K [45]). Due to this fact, the ignition temperature cannot be accurately measured and, thus, not discussed in this work. In spite of this, the changing tendency of the burning temperature can be well reflected by this profile.

Theoretically, the burning behavior of the Ti14 alloy is dominated by the burning of titanium since it is the major element of the alloy (Ti wt % > 85 wt %). In this regard, several chemical reactions would happen during the burning of titanium in air as summarized in Table 2. During the ignition period, the sample temperature increases gradually, and oxygen (from air) plays a crucial role (Reaction a). Afterwards, several reactions occur simultaneously (Reactions b–f), including the further melting of Ti (Reaction b) due to the intensive heat release from Reaction a, and the oxidation reaction between liquid Ti and O (Reaction c).

The two-stage burning process has been found in many burning processes of metals [41,42,43], and can be explained from the perspective of the controlling mechanisms, which is characterized by the ratio (ε) between the controlling chemistry kinetics (*R*_Kin_) and controlling oxygen transport (*R*_Tra_) [41], i.e., ε = *R*_Kin_*/R*_Tra_ = *A*/(*B* + *C*_o_). Here *A* is determined by a range of factors, including the reaction rate, burning radius, gas pressure and density, burning temperature, and diffusion coefficient. *B* is the quality stoichiometric coefficient. *C*_o_ is the oxygen partial pressure. During the ignition stage (i.e., ε >> 1), the oxygen diffusion rate is small, and the surface oxygen concentration is very high, which reveals that even the burning radius and burning temperature is small in ignition, the burning of the alloy was controlled by chemical reaction kinetics. Afterwards, in the stable burning stage, the burning process was majorly controlled by the transport of oxygen with ε << 1.

### 3.2. Ignition Stage

**Figure 4:**
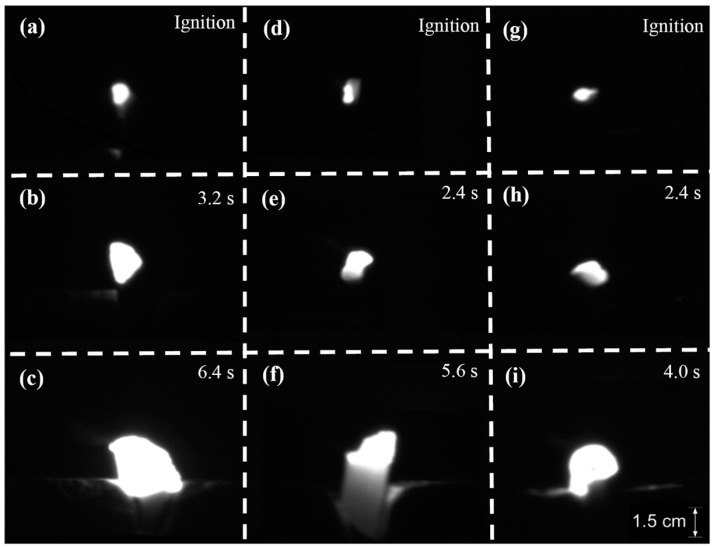
Captured images during burning for Ti14 alloy as obtained from: forging temperature of 1000 °C after ignition at the time of (**a**) 0.4; (**b**) 3.2; and (**c**) 6.4 s; forging temperature of 1050 °C after ignition at the time of (**d**) 0.4; (**e**) 2.4; and (**f**) 5.6 s; and forging temperature of 1100 °C after ignition at the time of (**g**) 0.4; (**h**) 2.4; and (**i**) 4.0 s. The oxygen partial pressure (*C*_o_) is 20% for all the three burning tests.

**Figure 5:**
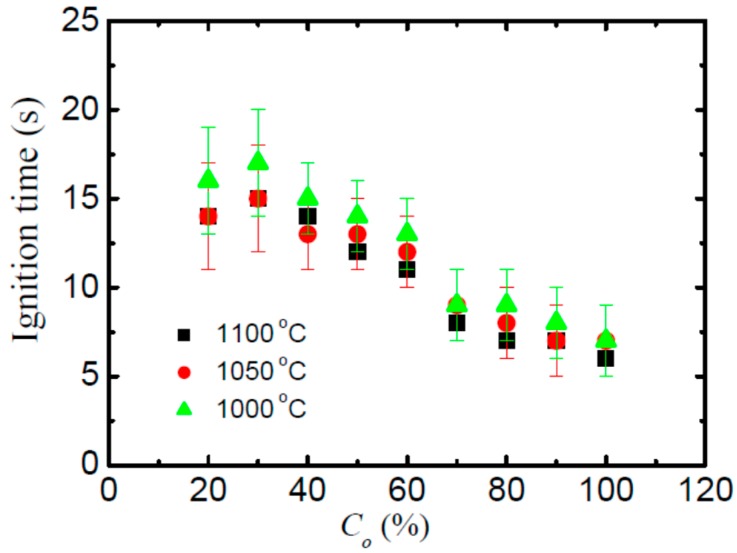
The ignition time as a function of the oxygen partial pressure (*C*_o_) for the Ti14 alloy as obtained from the forging temperature of 1000, 1050, and 1100 °C. The results are averaged over five burning tests. It is noticed that, to reduce the effect of heating times, a preheating process is provided to accelerate the ignition, which will influence the absolute ignition time, however, it will only influence the relative relationship between these values as focuses in this work.

The ignition stage can be characterized by brightness, which is caused by the complex oxidation of Cu and Ti in the alloy and is controlled by oxidation chemistry kinetics [46,47]. Ignition occurred when enough Ti reacts with oxygen to generate a visible flame on the sample surface (Figure 4). The increase of oxygen partial pressure (*C*_o_) in this experiment, even the gas pressures (*P*_o_) in laser ignition tests [36,44,47] and friction pressures (*P_f_*) in frictional ignition tests [40], will strengthen the burning chemistry kinetics and lead to shorter ignition time. In order to know the influence of oxygen partial pressure and forging sample on the burning chemistry kinetics, the ignition time where the time duration from heating to the detected ignition (from the recorded images) is performed as shown in Figure 5. As compared in Figure 5, the ignition time decreases generally with the increases of oxygen partial pressure for all samples. Shafirovich [48] and Molodetsky [45] reported that burning oxidation chemistry kinetics of titanium alloy in ignition is mainly dependent on the surface oxygen concentration and required reaction heat, while the addition of alloying element such as V, Cu, and Cr play an ignorably important role in determining the ignition time of the alloy, when the surface oxygen concentration and heat are sufficient. Similar results are also obtained in the ignition of Ti14 alloy, where burning chemistry kinetics exhibit high dependence on oxygen partial pressure. Overall, the forging temperature does not show a clear impact on the ignition time of the Ti14 alloy.

### 3.3. Stable Burning Stage

**Figure 6:**
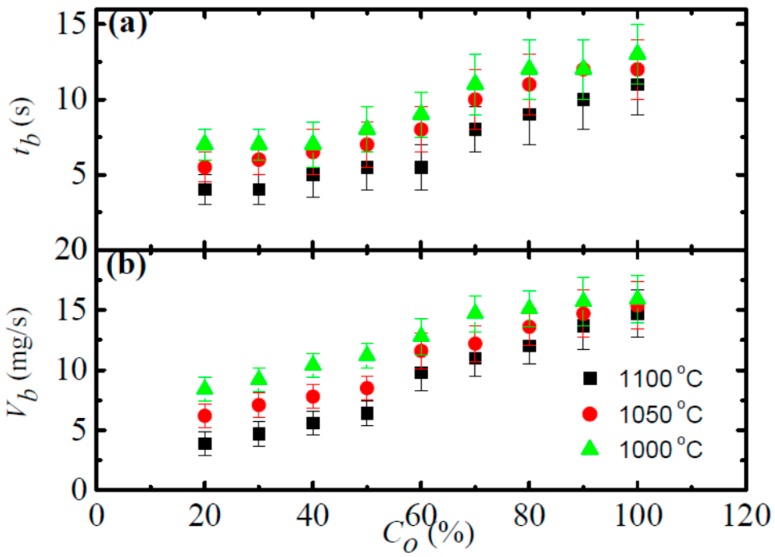
(**a**) The burning duration (*t_b_*) as a function of the oxygen partial pressure (*C*_o_); and (**b**) the corresponding burning velocity (*v_b_*) as a function of *C*_o_. The results are averaged over five burning tests.

As aforementioned, the stable burning stage of metal is controlled by oxygen transport and fast chemical reactions [40,41,42]. These chemical reactions will induce a rapid temperature climb (Figure 3) and cause high brightness (Figure 4). According to previous works [42,48], the stable burning stage can be characterized by the burning duration, burning velocity, and flame intensity. The burning velocity is estimated from *v_b_* = Δ*m_s_*/*t_b_*, with Δ*m_s_* representing the sample weight gain after burning and *t_b_* representing burning duration. In the meanwhile, the flame intensity can be either represented by the brightness or the flame height. In this work, flame height was estimated from the recorded images as shown in Figure 6. To mention that to ensure a reliable estimation, each burning test has been repeated five times with same burning setting up.

As illustrated in Figure 6a, the burning duration increases with the oxygen partial pressure (*C*_o_). Such a result is reasonable as higher *C*_o_ will enhance the burning process and lead to more sufficient burning, which will on the one hand increase the sample weight by oxidation reaction and on the other hand lead to longer burning duration [48]. From Figure 6b, it is found that the burning velocity shares a same increasing profile as that of the burning duration, which indicates that the increase of sample weight is more profound compared with that of the burning duration at higher *C*_o_. It is interesting that the higher forging temperature is intended to result in a shorter burning duration, as well as slower burning velocity in all tested oxygen partial pressures. For instance, the burning velocity (*v_b_*) at 20% *C*_o_ is around 7.8 mg/s (flow velocity of 15 m/s) for the Ti14 alloy obtained at 1000 °C forging temperature, which is two times higher than its counterpart forged at 1100 °C (~3.8 mg/s). In comparison, the as-casted Ti14 alloy is reported to have a *v_b_* of around 6.7 mg/s under the flow velocity of 10 m/s [49]. It is noticeable that although higher flow velocity would enhance the burning process (i.e., increase *v_b_*), the Ti14 alloy as obtained from forging temperature of 1050 and 1100 °C possesses much smaller *v_b_* (even under a higher flow velocity). Such obvious deviations among the *v_b_* are considered to be induced by the difference in microstructure and composition of the alloys [32], which vary vastly with the applied processing technique (i.e., casting or semi-solid forging) and processing parameters as forging temperature (Figure 1).

**Figure 7:**
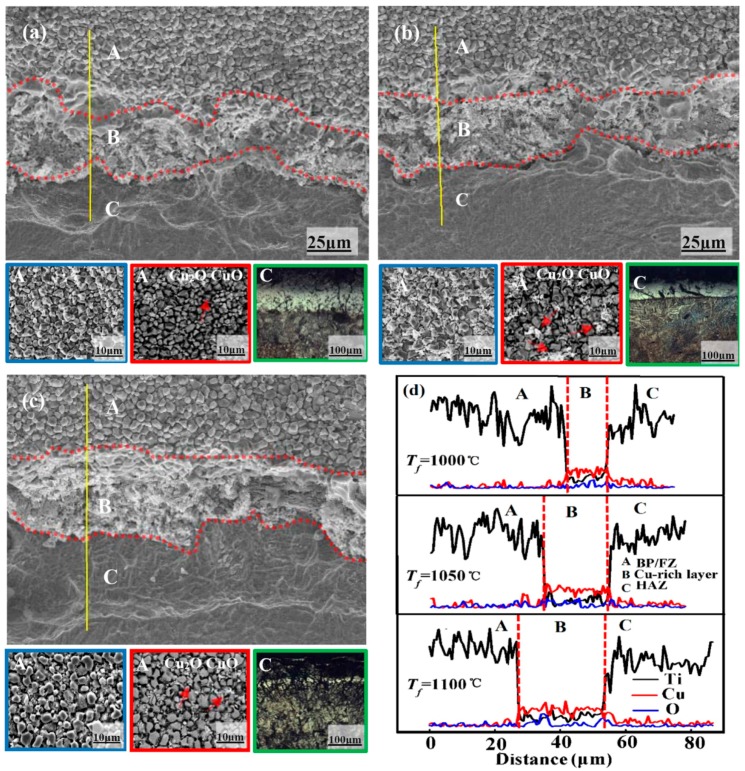
The SEM image of the burned Ti14 alloy as obtained from the forging temperature of: (**a**) 1000; (**b**) 1050; and (**c**) 1100 °C (*C*_o_ = 20%). A, B, C represent the BPZ/FZ, Cu-rich layer, and HAZ, respectively. The first two insets for each figure are the SEM (blue frame) and SEM-BSE (red frame) surface morphologies of the burning products, respectively. The last inset for each figure is the OM image (green frame), which shows the microstructure of Ti_2_Cu precipitates in HAZ from the corresponding Ti14 sample after burning; and (**d**) EDS of the burned structures which clearly show the distribution of Cu.

To reveal the results in such different burning behaviors, the cross-sectional morphologies of the burned sample at a typical oxygen partial pressure of 20% were compared. Similar with the results from that of Ti-V-Cr alloy [35,40] and TC4 alloy [49], it is identified an obvious burned product zone (BPZ), fusion zone (FZ), and heat affected zone (HAZ) for all examined samples (Figure 7a–c). Due to its easy fusion during burning, the burned product zone and fusion zone cannot be distinguished clearly from each other [25]. As illustrated in insets of Figure 7a–c, a typical burned surface morphology of titanium alloy with porous TiO_2_ particles was observed [40,50] (Figure 7a–c, blue and red frame). Such a porous structure acts as a transporting channel for oxygen during burning.

**Figure 8:**
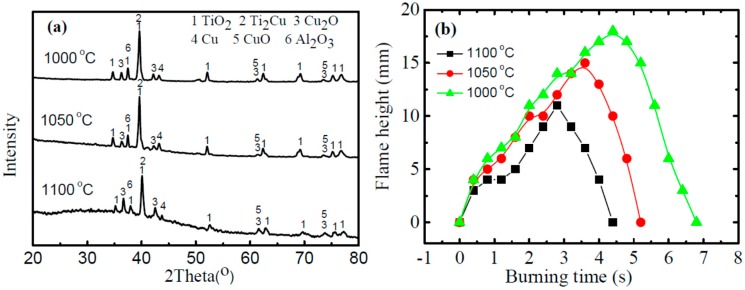
(**a**) XRD results of the burned products of forged Ti14 alloys (*C*_o_ = 20%); and (**b**) comparisons of the flame height as a function of the burning time for the Ti14 alloy obtained from different forging temperatures (*C*_o_ = 20%). The flame height was estimated from the recorded images every 0.4 s.

According to the XRD results (Figure 8a), there exists a mixture of CuO and Cu_2_O among the TiO_2_ particles in all samples. In addition, a certain amount of Ti_2_Cu and Al_2_O_3_ phases were also observed on the surface of the burned products. Specifically, the alloy obtained from higher forging temperature possesses more CuO and Cu_2_O phases after burning. To note no TiN phase is detected from the burned products, which is reasonable, as TiN will be further oxidized to TiO_2_ due to the high oxygen reactivity under air flow [51,52].

It is interesting that a clear Cu-rich layer was observed at the interface of BPZ/FZ and HAZ in all tested samples, as highlighted in Figure 7a–c (region B), which is not observed on the burn morphologies of Ti-V-Cr [35,40] and as-casted Ti-Cu burn resistant alloy [49]. The average thickness of Cu-rich layer increases with the forging temperature and a thickness of about 22 µm is obtained after being forged at 1100 °C, which is more than 50% larger than that after forged at 1000 °C (~14 µm). In particular, more Ti_2_Cu is found to precipitate and segregate to form the Cu-rich layer at higher forging temperature, which leads to thicker Cu-rich layer. It is supposed that this Cu-rich layer is behaving like a wall during the burning process, which will on the one hand reduce the exposure of titanium to oxygen, and on the other hand block the oxygen transportation channels [45,47,50]. In addition, the Cu-rich layer will reduce the intensity of oxidation reaction and hinder the burning of the alloy. Such assumption agrees with our experimental observations and the alloy forged at higher temperature exhibits smaller average flame height (or flame intensity) during the whole burning process, as compared in Figure 8b.

For the low thermal conductivity and oxidation exothermic reaction, the microstructure of the heat affected zone with different forging temperature is consisted of coarse grain and acicular Ti_2_Cu precipitates (Figure 7a–c green frame). It is observed that more Ti_2_Cu is precipitated and segregated to form a Cu-rich layer with higher forged temperature during burning, which is a benefit for the isolation of oxygen.

**Figure 9:**
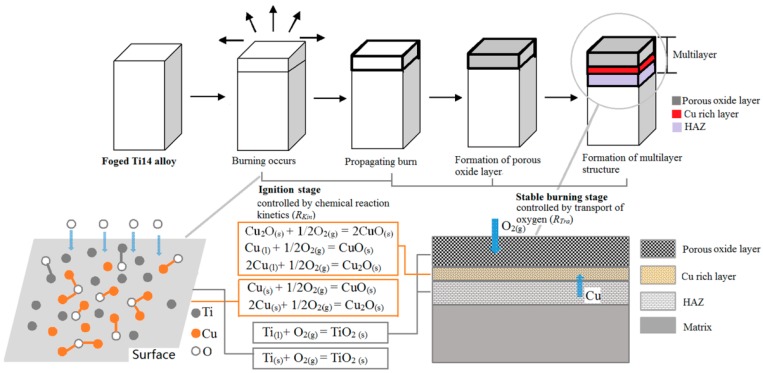
Schematic model showing the role of the Ti_2_Cu precipitates in the forged Ti14 alloy (as generated from the SSP) during the burning process.

The above discussion has clearly shown that the Ti_2_Cu precipitates, as generated by solid-state forging, greatly influences the burning behavior of the Ti14 alloy, and this role can be clearly seen from the schematic burning model, shown in Figure 9. It is well known that many factors are considered to affect the burning behavior of titanium alloy (such as heat, fuel, and metal burning reaction rates), the oxygen plays the most dominating roles [46]. The general results have shown that the burning of titanium is maintained by assimilating oxygen from the air and it is difficult to prevent the transport of oxygen (due to the porous structure after burning, i.e., the TiO_2_ particles). In this regard, the semi-solid forging provides a new avenue to enhance the burning resistance of the Ti14 alloy, through the generation of Ti_2_Cu precipitates in the alloy. In summary, the Ti_2_Cu precipitates will form a kind of oxygen isolation Cu-rich layer during burning, which improve the burning resistance of Ti14 alloy. Moreover, the Ti_2_Cu phase will melt before the burning, which will, on the one hand, consume part of the heat, and on the other hand, act as a lubricant on the surface to reduce the burning possibilities, especially under high-speed impact or friction.

## 4. Conclusions

Through a series of burning tests of the semi-solid forged Ti14 alloy, it is found that the semi-solid forging provides a new avenue to improve the burning resistance of the Ti14 alloy. The Ti_2_Cu phase precipitated during the semi-solid forging plays an important role in burning tests, since it could hinder the transport of oxygen in a stable burning stage through forming a kind of oxygen isolation Cu-enriched layer. Additionally, the Ti_2_Cu phase melts before burning, which consumes part of the heat (and reduces the alloy temperature), and may behave as a lubricant under dramatic impact and/or high-speed friction. These results suggest that the burn resistance of the Ti14 alloy can be effectively controlled through the semi-solid forging temperature, where the higher the forging temperature, the more Ti_2_Cu precipitates are obtained, i.e., the better burning resistance achieved. This may consummate the theoretical basis for the application of semi-solid processing technology of burn-resistant titanium alloy.

## Figures and Tables

**Table 1:** The relationship of the Ti_2_Cu fraction on grain boundary (*f_v_*), tensile strength, and ductility with the forging temperature for Ti14 alloy [33].

Properties	*f_v_* (%)	UTS (MPa)	∆UTS (%)	YS (MPa)	∆YS (%)	El (%)	∆El (%)
SF 950 °C	18	790	-	625	-	15	-
SSF 1000 °C	33	990	25.3	880	40.8	9	−40
SSF 1050 °C	35	970	22.3	855	36.8	8.5	−43.0
SSF 1100 °C	39	900	14.0	800	28	6.5	−56

Note: The Ti_2_Cu fraction on the grain boundary (*f_v_*) is estimated based on the qualitative metallography. SF and SSF represent conventional solid forging and semi-solid forging, respectively. UTS, YS, and EI denote the ultimate tensile strength, yield strength, and elongation. The relative changes of the mechanical properties of the alloy comparing with that obtained from SF are estimated as the following: ∆UTS (%) = (UTS_SSF_ − UTS_SF_)/UTS_SF_ × 100%, ∆YS (%) = (YS_SSF_ − YS_SF_)/YS_SF_ × 100%, and ∆El (%) = (El_SSF_ − El_SF_)/El_SF_ × 100%.

**Table 2:** Chemical reactions of titanium combustion in air [45].

Burning Stage	Chemical Reaction	Notation.
I	${\text{Ti}}_{\left(s\right)}+O_{2\left(g\right)}\to {\text{TiO}}_{2};\;∆H_{298}^{0}=-944\;\text{kJ}/\text{mol}$	a
II	${\text{Ti}}_{\left(s\right)}\to {\text{Ti}}_{\left(l\right)};\;∆H_{298}^{0}=+15\;\text{kJ}/\text{mol}$	b
	${\text{Ti}}_{\left(l\right)}+O_{2\left(g\right)}\to {\text{TiO}}_{2\left(s\right)};\;∆H_{298}^{0}=-929\text{kJ}/\text{mol}$	c
	$1/2{\text{Ti}}_{\left(l\right)}+1/2{\text{TiO}}_{2\left(s\right)}\to {\text{TiO}}_{\left(l\right)};\;∆H_{298}^{0}=-472\text{kJ}/\text{mol}$	d
	${\text{TiO}}_{2\left(s\right)}\to {\text{TiO}}_{2\left(l\right)};\;∆H_{298}^{0}=+67\text{kJ}/\text{mol}$	e
	${\text{Ti}}_{\left(l\right)}+1/2O_{2\left(g\right)}\to {\text{TiO}}_{\left(s\right)};\;∆H_{298}^{0}=-526\text{kJ}/\text{mol}$	f

Note: where Δ$H_{298}^{0}$ is room temperature standard enthalpy change of reaction. s, l, and g representing solid phase, liquid phase, and gas phase, respectively.
